# Skeletal muscle, haematological and splenic volume characteristics of elite breath-hold divers

**DOI:** 10.1007/s00421-019-04230-6

**Published:** 2019-09-21

**Authors:** Antonis Elia, Oliver J. Wilson, Matthew Lees, Paul J. Parker, Matthew J. Barlow, Matthew Cocks, John P. O’Hara

**Affiliations:** 1 Research Institute for Sport, Physical Activity and Leisure, Leeds Beckett University, Leeds, UK; 2 Division of Environmental Physiology, School of Chemistry, Bioengineering and Health, KTH Royal Institute of Technology, Stockholm, Sweden; 3 Research Institute for Sport and Exercise Sciences, Liverpool John Moores University, Liverpool, UK

**Keywords:** Apnoea, Skeletal muscle, Spleen, Myoglobin, Mitochondria, Haemoglobin

## Abstract

**Purpose:**

The aim of the study was to provide an evaluation of the oxygen transport, exchange and storage capacity of elite breath-hold divers (EBHD) compared with non-divers (ND).

**Methods:**

Twenty-one healthy males’ (11 EBHD; 10 ND) resting splenic volumes were assessed by ultrasound and venous blood drawn for full blood count analysis. Percutaneous skeletal muscle biopsies were obtained from the *m. vastus lateralis* to measure capillarisation, and fibre type-specific localisation and distribution of myoglobin and mitochondrial content using quantitative immunofluorescence microscopy.

**Results:**

Splenic volume was not different between groups. Reticulocytes, red blood cells and haemoglobin concentrations were higher (+ 24%, *p* < 0.05; + 9%, *p* < 0.05; + 3%, *p* < 0.05; respectively) and mean cell volume was lower (− 6.5%, *p* < 0.05) in the EBHD compared with ND. Haematocrit was not different between groups. Capillary density was greater (+ 19%; *p* < 0.05) in the EBHD. The diffusion distance (R_95_) was lower in type I versus type II fibres for both groups (EBHD, *p* < 0.01; ND, *p* < 0.001), with a lower R_95_ for type I fibres in the EBHD versus ND (− 13%, *p* < 0.05). Myoglobin content was higher in type I than type II fibres in EBHD (+ 27%; *p* < 0.01) and higher in the type I fibres of EBHD than ND (+ 27%; *p* < 0.05). No fibre type differences in myoglobin content were observed in ND. Mitochondrial content was higher in type I than type II fibres in EBHD (+ 35%; *p* < 0.05), with no fibre type differences in ND or between groups.

**Conclusions:**

In conclusion, EBDH demonstrate enhanced oxygen storage in both blood and skeletal muscle and a more efficient oxygen exchange capacity between blood and skeletal muscle versus ND.

## Introduction

Breath-hold diving is a competitive sport where maximal breath-hold time, or depth and distance swum underwater with a single breath is judged. The ability to suppress respiratory urges and attain long breath-hold durations is dependent on the collective contribution of, (1) the capacity for oxygen storage and utilisation, (2) training experience and (3) an individual’s psychological tolerance capacity to the increasing breathing urge. The lack of respiratory exchange at the lungs during voluntary breath-holding (apnoeas) necessitates reliance upon the body’s finite oxygen resources. The lung capacity, splenic volume and haematological characteristics (e.g. haematocrit and haemoglobin) of elite breath-hold divers (EBHD) have been examined extensively in the literature (Richardson et al. 2005; Ferretti et al. 2012; Schagatay et al. 2012; Fernadez et al. 2017). In comparison, relatively few studies have investigated the skeletal muscle characteristics of EBHD (Bae et al. 2003; Kjeld et al. 2018).

The oxygen storage and diffusion capacities of skeletal muscle may be important during periods of breath-hold diving and dynamic apnoeas where increased skeletal muscle recruitment and oxygen uptake occurs. The supply of oxygen to the mitochondria depends on connective transport in blood and on diffusive transport from blood to tissue (Schwerzmann et al. 1989; Pittman 2000; MacDougall and Sale 2014). In skeletal muscle, the distance that oxygen must diffuse from capillaries to skeletal muscle fibres depends on the capillarity of skeletal muscle (MacDougall and Sale 2014). Skeletal muscle capillary density can be increased after exercise training under hypoxic conditions (Terrados et al. 1990; Desplanches et al. 1993; Kon et al. 2014), and when exercise training sessions are accompanied by cold water immersion (D’Souza et al. 2018). Ama breath-hold divers perform between 113–138 short duration (< ~ 64 s) dives (5–25 m) per day, which can total up to 60% of their daily working period being submerged underwater in temperatures ranging from 10 °C to 24 °C (Hong et al. 1991; Schagatay et al. 2011). Bae et al. (2003) demonstrated that female Ama breath-hold divers have a higher skeletal muscle capillary density, a lower oxygen diffusion distance and a smaller muscle fibre cross-sectional area compared with a matched non-diving (ND) group. In addition, Park et al. (2005) failed to report any fibre type or capillarisation differences between control and habitual Indonesian breath-hold divers who dive all year round in moderate water temperatures (29–30 °C). Thus, the habitual divers’ skeletal muscle characteristics may be a chronic adaptive response to cold-water immersion, which may confer an advantage during their dives (Bae et al. 2003). To date the skeletal muscle capillarisation of the EBHD is unknown. EBHD have less frequent daily apnoeic activities (< 30 repetitions) than the Ama breath-hold divers, but perform repetitively longer apnoeas (> ~ 120 s) and attain deeper dives (> ~ 30 m) in temperatures ranging between 10 °C–28 °C . Whether these fundamental differences may stimulate skeletal muscle angiogenesis in EBHD skeletal muscles remains to be determined.

A high myoglobin concentration is regarded as an important adaptation to apnoeic diving in mammals and is closely related to their apnoeic and diving capabilities (Ponganis 2010). Myoglobin facilitates oxygen to the mitochondria during periods of increased metabolic activity and serves as an oxygen reservoir during times where ventilation stops and hypoxia ensues (Postnikova and Shekhovtsova 2013). Evidence suggest that skeletal muscle myoglobin expression and protein content increase in response to combined exercise and hypoxia (Terrados et al. 1990; Kanatous et al. 2009). However, Kjeld et al. (2018) found no between-group differences in myoglobin content after Western Blot analyses of skeletal muscle homogenates in the *m. vastus lateralis* of male EBHD compared with matched controls. Although Western Blot methodology is useful, it does not provide information on the localisation and spatial distribution of proteins nor the relative distribution of proteins between muscle fibre types. These challenges can be overcome using immunofluorescence microscopy where the localisation and (relative) distribution of proteins can be revealed and quantitated as they occur in human skeletal muscle in situ.

It is well accepted that normoxic training stimulates mitochondrial biogenesis and augments mitochondrial content (Gollnick et al. 1972; Burgomaster et al. 2008; Gibala, 2009). Conversely, hypoxic training has been shown to impair these adaptive mitochondrial responses (Mizuno et al. 1990; Zhang et al. 2008; Bakkaman et al. 2007). Bakkaman et al. (2007) reported a significant increase in citrase synthase following 4 weeks of one leg exercise in normobaric conditions, whereas no differences were observed under simulated hypoxic training. This blunted training response in muscle oxidative potential during hypoxia may be advantageous in a breath-hold diving context. Interestingly, a lower mitochondrial oxygen consumption during non-phosphorylating and uncoupled respiration was identified in the *m. vastus lateralis* of male EBHD compared with matched controls (Kjeld et al. 2018). Therefore, the lower mitochondrial respiratory capacity may be important for oxygen conservation during prolonged dynamic breath-hold attempts. Currently, little is known about the effect of apnoeic training on fibre type-specific mitochondrial content and cellular distribution. Accordingly, this study will examine for the first time the EBHD *m. vastus lateralis* mitochondrial content and relevant localisation and fibre-type distribution.

To date, no study has collectively examined the splenic volumes, haematological and skeletal muscle characteristics of EBHD. The integration of these physiological systems may reveal important chronic adaptations and/or compensations which may advance our current understanding of the oxygen storage, delivery and exchange capacities of EBHD and ultimately provide further insight to their apnoeic capabilities. Therefore, the purpose of this study was to (1) examine the skeletal muscle capillarisation, fibre type-specific protein content of myoglobin and mitochondria in EBHD compared with a ND group using immunofluorescence microscopy and (2) provide a novel integrated analysis of the EBHD splenic volumes, haematological and skeletal muscle characteristics and compare them with a ND group. The present study tested the hypothesis that EBHD would combine a higher oxygen storage and a more efficient oxygen exchange capacity compared with ND.

## Materials and methods

### Participants

Eleven male EBHD and ten male ND volunteered for this study. EBHD had 7 ± 2 years of apnoea practice and their training regime consisted of 8 ± 2 hours per week  of apnoea related activities (Table 1). Additionally, all EBHD were national team members, of which 1 was a current world champion, 4 were current and 2 former national record holders. The ND were physically active individuals and had no prior breath-hold diving experience. Participants were healthy, non-smoking, habitual sea-level residents and provided written informed consent before the study. All experimental procedures were completed in accordance with the ethical standards of the Leeds Beckett University ethical committee (ethical approval number 52330) and with the Declaration of Helsinki.

**Table 1:** Mean (± SD) participant characteristics

Variables	ND (*n* = 10)	EBHD (*n* = 11)
Height (m)	1.82 ± 0.1	1.80 ± 0.1
Body mass (kg)	85 ± 7	82 ± 11
Static apnoeas (s)	N/A	414 ± 101
Dynamic apnoeas with fins (m)	N/A	202 ± 46
Dynamic apnoeas without fins (m)	N/A	145 ± 50

### Experimental protocol

Following arrival at the laboratory, the participant’s anthropometric measurements, including height and body mass were recorded (Table 1). EBHD were anthropometrically matched to ND.

### Splenic volume measurements

Participants completed 20 min of supine rest following which their resting splenic volumes were assessed using a non-invasive ultrasonic portable device (MindRay DP-50, Shenzhen MindRay Bio-Medical Electronics Co., Ltd., Shenzhen, China). Participants were seated vertically while the site for spleen measurements was identified from the dorsal side. Thereafter, three measurements of each triaxial measurement point of the spleen’s maximal length, thickness and width were determined, with the mean for each point being used to calculate splenic volume through the use of the Pilström formula (*Lπ*[*WT* − *T*^2^]/3) (Schagatay et al. 2012).

### Blood analysis

One whole blood sample (4 mL; BD Vacutainer, K2E EDTA, BD, Plymouth, UK) was drawn from a suitable vein in the antecubital fossa of the participant’s arm (median cubital vein and basilic vein) for a full blood count analysis (reticulocytes, red blood cells, haemoglobin, haematocrit and mean cell volume) to be performed (Advia 2120i Haematology System, Siemens Healthcare, Surrey, UK).

### Muscle sample

Following an additional 30 min of supine rest one percutaneous skeletal muscle biopsy was performed under local anesthesia with bupivacaine hydrochloride (0.5%) from the *m. vastus lateralis* of each participant using a standard Bergstrom needle muscle biopsy technique adapted with suction (Tarnopolsky et al. 2011). Samples were blotted to remove excess blood, with visible connective tissue and fat removed from the muscle samples. Samples were then mounted with muscle fibres in transverse orientation on triangular cork blocs in Tissue-Tek OCT compound and were then frozen in liquid nitrogen-cooled isopentane and stored at – 80 °C until analysis.

### Immunofluorescence staining

Duplicate 5 µm thick serial cryosections of skeletal muscle were sliced at − 20 °C (CM1860, Ag Protect Leica Biosystems, UK). Care was taken to ensure the skeletal muscle fibres were orientated in the transverse plane. Muscle cross-sections from EBHD and ND were positioned alongside each other on uncoated glass slides (VWR International, Leicester, UK) and left to air-dry (~5 min) at room temperature prior to treatment. Muscle cross-sections were fixed for 5 min in an acetone and ethanol (3:1) solution and were then washed 3 × 5 min in a phosphate buffered saline (PBS) solution (7.4 pH). Thereafter, 25 µL of normal goat serum (10%) was applied to each muscle cross-section and were incubated for 10 min and washed 3 × 5 min in PBS. Cross-sections were co-incubated with either anti-myoglobin (Abcam; cat. no. ab77232) at a 1:200 dilution or anti-cytochrome oxidase IV (ThermoFisher Scientific; cat. no. 1D6E1A8) at a 1:100 dilution for the determination of myoglobin and mitochondrial content, respectively. Anti-dystrophin (Sigma-Aldrich; cat. no. D8168) at a dilution 1:400 was used as a marker for the muscle fibre plasma membrane and a 1:100 dilution of anti-myosin heavy chain type I (Developmental Studies Hybridoma Bank; cat. no. A4.840) was used to identify type I skeletal muscle fibres leaving type II fibres unstained. Samples were incubated at room temperature for 2 hours. Following primary antibody incubation, sections were washed 3 × 5 min in PBS and then incubated for 30 min at room temperature in 1:200 dilutions of the appropriately labelled fluorescent conjugated secondary antibodies. Alexa Fluor 594-conjugated goat anti-rabbit IgG was used to detect anti-myoglobin. Alexa Fluor 594-conjugated goat anti-mouse IgG2a was used to detect anti-Cytochrome oxidase IV. Alexa Fluor 488-conjugated goat anti-mouse IgG2b was used to detect anti-dystrophin. Alexa Fluor 350-conjugated goat anti-mouse IgM secondary antibody was used to detect anti-myosin heavy chain type I. Capillaries were identified by staining muscle cross-sections with Fluorescein isothiocyanate (FITC)-conjugated anti-Ulex Europaeus Agglutinin-I (UEA-I) (Sigma-Aldrich; cat. no. L9006). After incubation with the secondary antibodies, muscle cross-sections were washed 3 × 5 min in PBS. Once dry, glass cover slips were mounted in a glycerol and mowiol 4–88 solution in 0.2 m Tris buffer (pH 8.5) with addition of 0.1% DABCO anti-fade medium.

### Antibody specificity

**Fig. 1:**
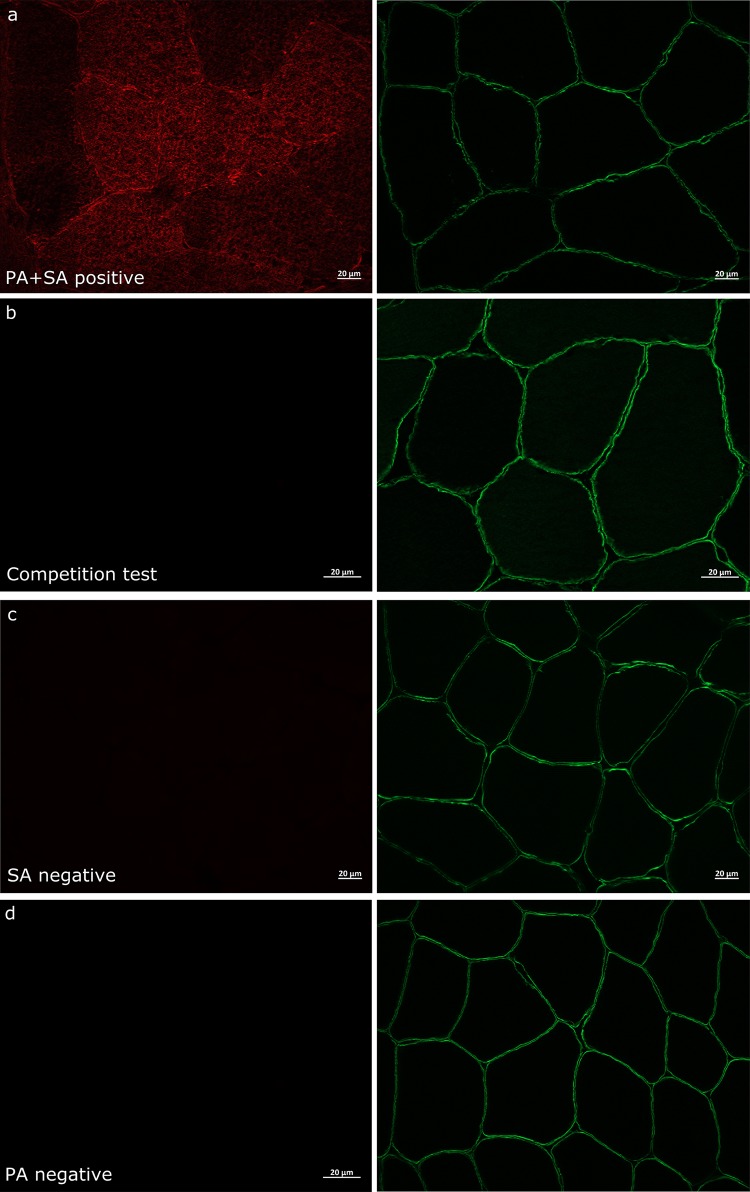
Specificity of myoglobin antibody used for immunofluorescence analysis using anti-myoglobin blocking peptide and double negative controls. Immunofluorescence detection of myoglobin (red) in human skeletal muscle after pre-incubation of the anti-myoglobin antibody in the absence (**a**) or presence (**b**) of a specific blocking peptide. **c** Negative control where muscle cross-sections were incubated with anti-myoglobin in the absence of the appropriate secondary antibody. **d** Negative control where muscle cross-sections were incubated with the secondary antibody in the absence of anti-myoglobin. The cell membrane is stained with anti-dystrophin (green). Scale bars 20 µm.

Antigen specificity and selectivity of the myoglobin antibody was confirmed by the absence of positive immunofluorescence signal when anti-myoglobin was preincubated overnight at 4 °C with a 10 × concentrated myoglobin-specific blocking peptide (Santa-Cruz, cat. no. sc-393020) (Fig. 1a, b). Double negative control experiments were also performed, where the staining protocol was completed following the same procedure as positive experiments, but either the anti-myoglobin or the anti-myoglobin-specific secondary antibody was omitted from the PBS solution (Fig. 1c, d). In both experiments, a positive signal was absent.

### Reproducibility of quantitative immunofluorescence

Immunofluorescence analysis of myoglobin content was performed in triplicate. The coefficient of variation (CV) for the intramuscular myoglobin content was 5.1%.

### Image capture

Slides were viewed using a ZEISS AxioImager M2 automated microscope with Axiocam 506 mono camera (ZEISS, Gottingen, Germany). An A-Plan 20 ×/0.45 numerical aperture Ph2 objective was used to capture images for the quantification of fibre type distribution and capillarisation. A Plan-Apochromat 40 ×/1.3 numerical aperture oil Ph3 [UV] VIS-IR objective (Gottingen, Germany) was used with Apotome.2 structured illumination to capture detailed images of myoglobin and mitochondrial content. Identical camera settings were used to capture all images between participants on the same glass slide. The 4′-6-diamidino-2-phenylindole (DAPI) UV (340–380 nm) excitation filter was used to visualise Alexa Fluor 350 fluorophores (blue; myosin heavy chain I within type I muscle fibres), the FITC (465–495 nm) excitation filter was used to visualise Alexa Fluor 488 fluorophores (green; dystrophin and capillaries), and the Texas-Red (540–580 nm) excitation filter was used to visualise Alexa Fluor 594 fluorophores (myoglobin and COXIV as a marker of the mitochondria). For the duplicate cross-sections, 10 images were captured per cross-section (totalling 20 per participant).

### Image analysis

Image processing was completed using Image J Fiji Version 1.0 software. Muscle fibre cross-sectional area was determined either through the use of an automated method or where necessary it was performed manually by tracing along the dystrophin-stained border. Only whole and transversely orientated skeletal muscle fibres (fibre roundness of  < 1.639) were analysed (Kosek et al. 2006).

Skeletal muscle fibre type distribution and capillarisation were quantified from 1505 transversely orientated skeletal muscle fibres of which 721 fibres were from EBHD and 784 fibres were from ND. Capillarisation was quantitated using the following indexes (1) capillary density, (2) fibre type distribution, (3) fibre area (FA), (4) fibre perimeter, (5) capillary-to-fibre perimeter exchange index (CFPE index), (6) capillary contacts (CC), (7) capillary-to-fibre ratio (C:F_i_), (8) diffusion distance (R_95_), (9) capillary supply (CS) and (10) sharing factor (SF) (Hepple 1997; Hepple et al. 1997). Fibre cross-sectional area and perimeter were measured on calibrated images using Image J Fiji Version 1.0 software. Myoglobin immunofluorescence signal intensity was quantified from a total of 1374 transversely orientated muscle skeletal fibres of which 783 fibres were from EBHD and 591 fibres were from ND. Fibre type-specific myoglobin content was determined from a mean of 50 ± 21 type I and 29 ± 13 type II fibres in EBHD and from 45 ± 18 type I and 29 ± 13 type II fibres in ND. Mitochondria immunofluorescence signal intensity was quantified from a total of 1238 transversely orientated muscle skeletal fibres of which 626 fibres were from EBHD and 612 were from ND. Fibre type-specific mitochondrial content was determined from a mean of 53 ± 19 type I and 36 ± 8 type II fibres in EBHD and from 38 ± 16 type I and 49 ± 15 type II fibres in ND.

### Statistical analysis

All data were statistically analysed using the IBM SPSS statistics software version 21. The Shapiro–Wilk test was used to assess normality, whereas homogeneity was assessed using Levene’s test. Paired sample *t* tests were used to assess within group differences and independent sample *t* tests were used to assess between group differences. Pearson’s correlation was utilised to assess the relationship between splenic volume, height and body mass. Data were reported as means ± SD and significance was accepted at *p* < 0.05. GraphPad Prism version 7.0 was used to construct figures.

## Results

### Splenic volumes

Splenic volumes (Table 2) ranged between 128 and 499 mL in the EBHD and 198–452 mL in the ND. There were no significant differences in resting splenic volumes between groups (Table 2). No relationship was observed when splenic volume was correlated to height (*r* = − 0.0001, *p* = 0.995) or body mass (*r* = − 0.040, *p* = 0.863) for either group.

**Table 2:** Mean (± SD) splenic volume and haematological characteristics.

Variables	ND (*n* = 10)	EBHD (*n* = 11)	*p* values
Spleen volume (mL)	297 ± 77	300 ± 122	0.947
Reticulocytes (%)	1.13 ± 0.30	1.47 ± 0.29	0.019
Red blood cells (10^12^/L)	4.93 ± 0.24	5.43 ± 0.49	0.015
Haemoglobin (g/L)	148 ± 3	153 ± 6	0.039
Haematocrit (%)	45 ± 2	46 ± 3	0.361
Mean cell volume (fl)	92 ± 4.33	86 ± 6.64	0.044

### Haematological markers

Reticulocytes were significantly higher (24%) in the EBHD (range 1.1–2.0%) compared with the ND (range 0.7–1.60%) (Table 2). Red blood cell count and haemoglobin concentrations were significantly higher in the EBHD (9%; 3%, respectively) compared with the ND and ranged between 4.80–6.3·10^12^/L and 144–161 g/L for the EBHD, 4.64–5.34·10^12^/L and 145–152 g/L for the ND (Table 2). Resting mean cell volume was 6.5% significantly lower in the EBHD compared with the ND and ranged between 70–93 fl in the EBHD and between 87–97 fl in the ND (Table 2). No group differences were identified in resting haematocrit with concentrations ranging between 42–52 % in the EBHD and between 43–49 % in the ND (Table 2).

The resting haematocrit concentration of an EBHD was 52%, this ranges above the recommended limits for athletic competitions (Cazzola 2000). However, similar observations were made by Johansson et al. (2009) that reported a high haematocrit concentration (51%) in 1116 out of 36,962 healthy male blood donors. Additionally, the EBHD resting reticulocytes (1.1%), red blood cell count (5.2·10^12^/L), haemoglobin (155 g/L), erythropoietin (5.79 ml U/mL), iron (14 μmol/L) and ferritin (82 μg/L) concentrations were within the normal range expected for healthy male adults, providing further evidence that the EBHD was not under the influence of doping.

### Skeletal muscle characteristics

Capillary density was significantly higher (19%) in EBHD compared with ND (Table 3). There was no difference in fibre type distribution between groups (Table 3). Type I mean FA and perimeter was significantly lower (24%) than type II fibres only in the ND group, with no between group differences (Table 3). Both groups had a significantly greater CFPE index in their type I muscle fibres compared with type II fibres, but no between group differences were observed (Table 3). CC were similar across fibre types and between groups. Overall C:F_i_ was only significantly higher (18%) in the EBHD group compared with ND, with no C:F_i_ differences reported within groups. A lower R_95_ and CS were reported in type I muscle fibres compared with type II fibres in both groups (Table 3). When type I fibres R_95_ was compared between groups, EBHD had a significantly lower (13%) diffusion distance compared with the ND group (Table 3), whereas no group differences were reported in CS muscle fibres. A significantly lower (3%) overall SF was observed in the EBHD compared with the ND group, whereas no within fibre type group differences were observed (Table 3).

**Table 3:** Mean (± SD) capillarisation measurements

Variable	ND	EBHD
Capillary density (capillaries/mm^2^)	490 ± 99	605 ± 87**
Type I distribution (%)	55 ±13	56 ± 12
Type II distribution (%)	45 ± 13	44 ± 12
Overall FA (μm^2^)	5163 ± 804	5575 ± 805
Type I FA (μm^2^)	4596 ± 942*	5233 ± 1196
Type II FA (μm^2^)	6082 ± 1326	5750 ± 677
Overall perimeter (μm)	298 ± 24	314 ± 28
Type I perimeter (μm)	281 ± 31*	305 ± 38
Type II perimeter (μm)	326 ± 36	324 ± 36
Overall CFPE Index (capillaries·1000 μm^−1^)	6.40 ± 0.90	7.06 ± 0.87
Type I CFPE Index (capillaries·1000 μm^−1^)	6.92 ± 0.88*	7.61 ± 0.89*
Type II CFPE Index (capillaries·1000 μm^−1^)	5.81 ± 0.80	6.27 ± 0.74
Overall CC	5.21 ± 0.55	5.92 ± 0.92
Type I CC	5.34 ± 0.83	6.17 ± 1.10
Type II CC	5.19 ± 0.48	5.49 ± 0.81
Overall C:F_i_	1.89 ± 0.23**	2.23 ± 0.40
Type I C:F_i_	1.95 ± 0.34	2.34 ± 0.49
Type II C:F_i_	1.87 ± 0.20	2.04 ± 0.33
Overall R_95_ (μm^2^)	33.89 ± 5.35	29.85 ± 3.53
Type I R_95_ (μm^2^)	31.16 ± 3.84*	27.59 ± 3.10*^,^**
Type II R_95_ (μm^2^)	37.03 ± 6.14	33.57 ± 4.42
Overall CS (μm^2^)	2999 ± 865.62	2679 ± 343.10
Type I CS (μm^2^)	2570 ± 551.37*	2333 ± 220.81*
Type II CS (μm^2^)	3537 ± 1146	3092 ± 485.09
Overall SF	2.83 ± 0.04**	2.76 ± 0.07
Type I SF	2.83 ± 0.06	2.73 ± 0.09
Type II SF	2.84 ± 0.05	2.77 ± 0.07

*FA* fibre cross-sectional area, *CFPE index* capillary-to-fibre perimeter exchange index, *CC* capillary contacts, *C*:*F*_*i*_ capillary-to-fibre ratio, *CS* capillary supply, *R*_95_ diffusion distance, *SF* sharing factor

Significance is denoted as *within group fibre type differences, **between groups (*p* < 0.05)

**Fig. 2:**
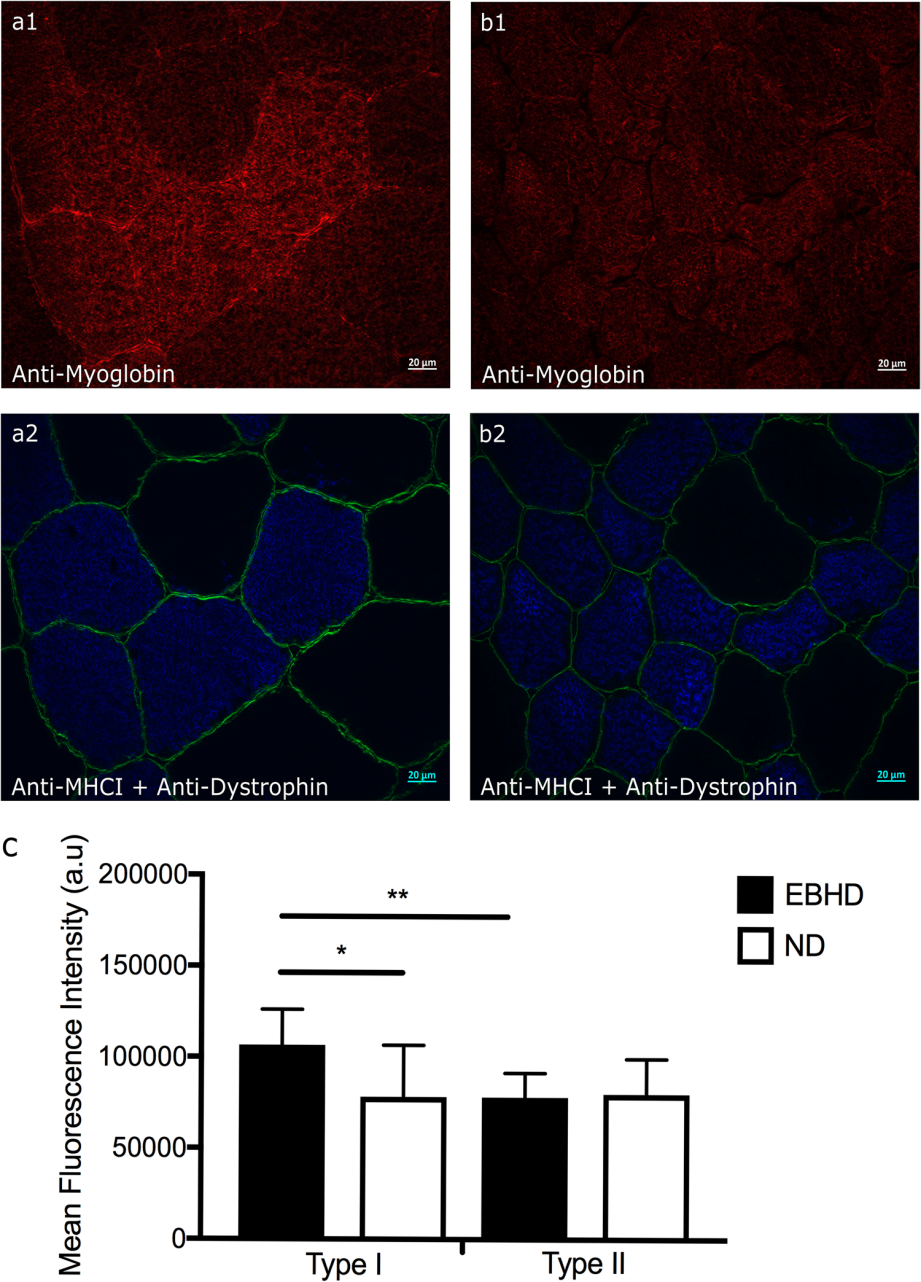
Representative widefield fluorescence microscopy images anti-myoglobin (red), co-incubated with anti-myosin heavy chain type I (blue) for identification of type I fibres and anti-dystrophin (green) for identification of the sarcolemma, in resting human *m. vastus lateralis*. Representative widefield microscopy images of myoglobin in transversely orientated type I and type II muscle fibres of an EBHD (**a1**) and a ND (**b1**). **c** Fibre type-specific mean immunofluorescence intensity of myoglobin in EBHD and ND. Significance denoted as *p = 0.025 for between group differences and **p = 0.004 for within group differences. Scale bars 20 µm.

The anti-myoglobin antibody successfully stained the muscle fibres. The myoglobin stains were only visualised when samples were incubated with the anti-myoglobin primary antibody as well as with the corresponding secondary antibody conjugated with fluorescence (Fig. 1a). Antigen specificity and selectivity of the myoglobin antibody was also confirmed by the absence of positive immunofluorescence signal when, (1) anti-myoglobin was pre-incubation with a myoglobin-specific blocking peptide (Fig. 1b), and (2) when samples were incubated with either the anti-myoglobin primary antibody (Fig. 1c) or the anti-myoglobin-specific secondary antibody (Fig. 1d). These experiments indicated that the anti-myoglobin antibody specifically recognises myoglobin protein content in human skeletal muscle *in vivo*.

Myoglobin content was significantly higher (27%) in type I versus type II muscle fibres in EBHD (*p* = 0.004), whereas no fibre type differences were observed in ND (*p* = 0.783) (Fig. 2). Myoglobin content was significantly greater (27%) in the type I fibres of EBHD compared with the type I fibres of ND (*p* = 0.025), but there was no between-group differences in type II fibre myoglobin content (*p* = 0.805) (Fig. 2). Qualitatively assessing the localisation of myoglobin content of both groups we observed that EBHD have a stronger fluorescence signal closer to the sarcolemma region of type I muscle fibres, with a homogenous distribution at the subsarcolemmal region (Fig. 2a1).

**Fig. 3:**
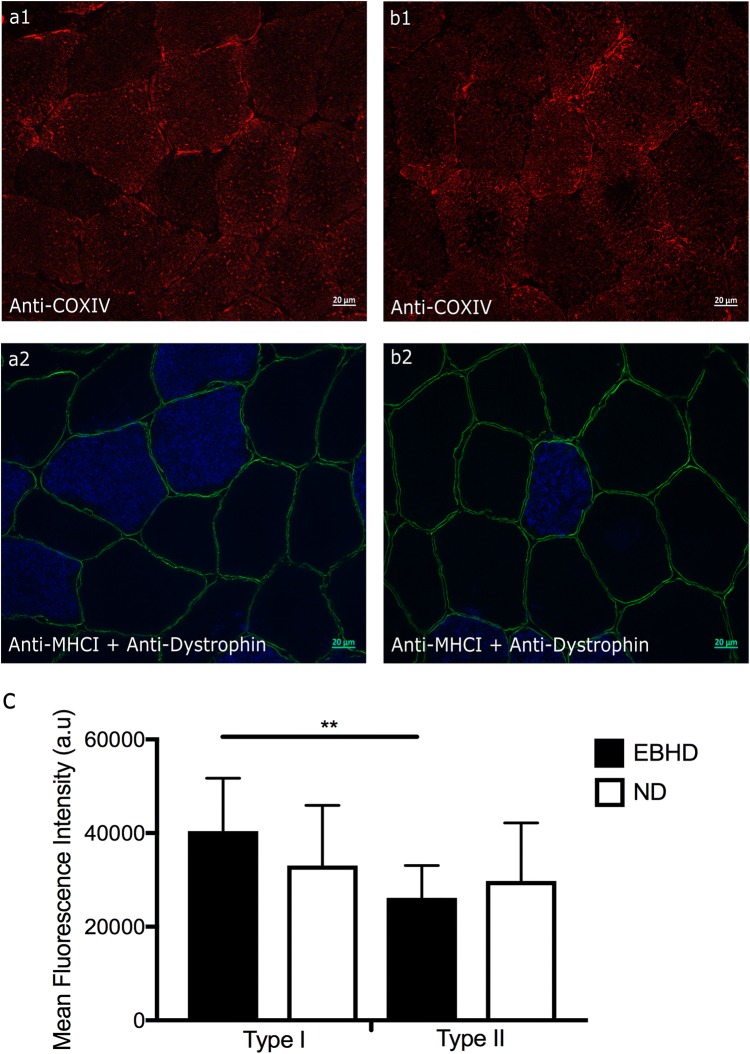
Representative widefield fluorescence microscopy images of anti-COXIV for visualisation of the mitochondria co-incubated with anti-myosin heavy chain type I (blue) for muscle fibre type differentiation and anti-dystrophin (green) for identification of the sarcolemma in resting human *m. vastus lateralis*. Representative widefield microscopy images of mitochondria in transversely orientated type I and type II muscle fibres of an EBHD (**a1**) and a ND (**b1**). **c** Fibre type-specific mean immunofluorescence intensity of mitochondria in EBHD and ND. Significance denoted **p = 0.004 for within group differences. Scale bars 20 µm.

Mitochondria content was significantly higher (35%) in the type I muscle fibres compared with type II fibres in the EBHD (*p* = 0.004), whereas no fibre type difference was observed in the ND (*p* = 0.38) (Fig. 3). There were no between-group differences in fibre type-specific mitochondrial content (type I, *p* = 0.277; type II, *p* = 0.515) (Fig. 3). Qualitatively assessing the localisation of mitochondrial content, we observed that EBHD have a stronger fluorescence signal closer to the sarcolemma region of type I muscle fibres (Fig. 3a1) with a homogenous distribution at the subsarcolemmal region. In the ND group, we observed a more heterogeneous subsarcolemmal distribution of mitochondrial content with a tendency for a lower fluorescence signal at the deeper areas (centre) of the myofibres (Fig. 3b1).

## Discussion

The primary findings were that EBHD; (1) combined a splenic volume that was not different to ND with a greater red blood cell and haemoglobin concentration; (2) had a greater capillarity and improved R_95_ of skeletal muscle which supplied; (3) a greater type I fibre myoglobin content and a mitochondrial content that was not different to ND. These findings confirm our hypothesis that EBHD have a greater oxygen reserve and a more efficient oxygen exchange capacity between blood and skeletal muscle and that this may, at least in part, contribute to the greater breath-holding capacities reported in this population.

Splenic volumes were not different between EBHD and ND and there was no relationship between splenic volume and height or body mass. These observations are in agreement with Schagatay et al. (2012) and provide further evidence that splenic volumes are not related to an individual’s height or body mass (Prassopoulos et al. 1997). A larger splenic volume with the capacity to store a greater amount of erythrocytes is considered advantageous in an apnoeic context, with positive correlations reported between an individual’s apnoeic capabilities and splenic volume (Schagatay et al. 2012). Although, in line with the literature we failed to report any splenic volume differences between diving and non-diving populations (Bakovic et al. 2003; Prommer et al. 2007), interestingly, the splenic volumes of five EBHD and two ND recruited in the current study spanned above (383–499 mL) the range of splenic volumes reported in 185 healthy adults (107–342 mL) (Prassopoulos et al. 1997; Lingurary et al. 2013). Evidence suggests that splenic size is partly governed by a genetic predisposition, a natural selection on genetic variations in the PDE10A gene, resulting in an increased splenic volume (Ilardo et al. 2018). Therefore, the splenic volume discrepancies reported amongst our diving and non-diving groups may, at least in part, be explained by the findings of Illardo et al. (2018).

A higher red blood cell concentration was observed in the EBHD, which spanned the upper range of red blood cell concentrations (3.80–5.50·10^12^/L) reported in 1258 age-matched healthy male adults (Osei-Bimpong et al. 2012). The higher haemoglobin concentrations in EBHD, are in agreement with previous observations in EBHD (Richardson et al. 2005; Fernadez et al. 2017). A high haemoglobin concentration is a beneficial adaptation to apnoeic diving in marine mammals (Ponganis 2011), as its upregulation enhances the oxygen transport capacity of blood to the recruited skeletal muscles and vital organs. These higher red blood cell and haemoglobin concentrations may be related to the nature of the breath-hold diver’s habitual training regime (transient acute hypoxia, spaced by periods of normal breathing). Kjeld et al. (2015) observed a 24% increase in erythropoietin 3 h after a maximal static and dynamic apnoea attempt. Thus, as erythropoietin is a key regulator of erythropoiesis (Jelkmann 2011), the higher reticulocytes and red blood cells reported in the EBHD might be the result of a chronic adaptation to repeated hypoxic exposures. However, future research should aim to examine the longitudinal effects of apnoeic training to test this hypothesis.

To the best of our knowledge, the present study is the first to report the mean cell volume of EBHD. The EBHD mean cell volume spanned the lower range (83–101 fl) observed in age-matched healthy male adults (Osei-Bimpong et al. 2012). The lower mean cell volume in EBHD versus ND suggests that the total surface area of the red blood cells is larger in EBHD. Holland and Forster (1966) demonstrated that cell volume is an important morphological factor that influences the oxygen transfer efficiency of red blood cells, with the initial rate of oxygen uptake by red blood cells varying inversely with cell volume (Holland and Forster 1966). Therefore, a larger red blood cell volume increases the intracellular diffusion path with a concomitant decrease in the permeability of the red blood cell membrane. Additionally, Vandergriff and Olson (1984) demonstrated that oxygen release was less dependent on red blood cell size and shape than oxygen uptake. Thus, the lower mean cell volume reported in the EBHD group might be advantageous, as haemoglobin will have a higher surface area to bind oxygen. Additionally, the lower mean cell volume of EBHD, coupled with the similar haematocrit concentrations reported across groups may upregulate the oxygen transport capacity of blood to recruited skeletal muscles in EBHD. Interestingly, a lower mean cell volume was also reported in Chilean and Nepalese altitude natives (3700 m), (85 fl and ~ 83 fl, respectively) (Wu et al. 2005). Taken together, these findings suggest an important role for hypoxia in promoting adaptations within the circulatory system for augmented oxygen storage and diffusion.

We made the first investigations of the skeletal muscle phenotypes of male EBHD using an immunofluorescence microscopy technique. Utilising a statistical approach consistent with previous literature in the area (Hepple et al. 1997; Bae et al. 2003; Park et al. 2005), a higher capillary density, C:F_i_ and a lower R_95 _was observed in the skeletal muscle of EBHD versus ND which suggests an enhanced blood to skeletal muscle fibre exchange capacity (Saltin 1985; Richardson et al. 1994). Firstly, for any given skeletal muscle blood flow, the greater capillary density will lead to longer mean transit time of red blood cells, thereby facilitating an extended period for oxygen exchange (Andersen and Saltin 1985; Richardson et al. 1999). Additionally, a higher capillary density increases the surface area for diffusion and decreases the overall diffusion distance between tissue and blood (Krogh 1919; Richardson et al. 1993), allowing for an improved diffusive exchange capacity (Saltin 1985; Richardson et al. 1994). As a result, the rate at which oxygen enters the muscle fibre and by-products are removed from the fibre during exercise is accelerated (Tesch et al. 1981; Tesch and Wright 1983; McGuire and Secomb 2003). Therefore, the higher capillary density observed in the EBHD is an advantageous adaptation for gas exchange at the tissue level due to a reduction in mean transit time of red blood cells and oxygen diffusion distance.

A lower overall SF was identified in our EBHD compared with ND (Table 3). Evidence suggests that the sharing of capillaries by more than a single fibre implicates the size of the domain that is supplied by a given capillary (Kreuzer et al. 1991). Thus, for a similar FA (between groups), the lower SF observed in the EBHD entail that the capillaries supplying a single fibre are shared across less fibres—indicative of an improved diffusive exchange capacity between tissue and blood. Our capillarisation data (lower R_95_ and higher capillary density) are in agreement with those previously reported in female Ama breath-hold divers (Bae et al. 2003). The greater capillarisation observed in EBHD compared with ND may be attributed to their habitual apnoeic training, where periods of static and dynamic apnoeas are frequently repeated. It is well accepted that hypoxia serves a vital role in the regulation and expression of vascular endothelial growth factor (Breen et al. 1996; Gustafsson and Sundberg 2000), and consequently, the initiation of capillary neo-formation and angiogenesis (Arany et al. 2008). Therefore, the greater capillarisation observed in EBHD may be attributed to the interaction between hypoxia and muscle recruitment occurring during apnoeic training.

No between group differences in fibre type distribution (type I vs type II) were observed in the present study. This is consistent with earlier observations made in EBHD and swimmers (Gollnick et al. 1972; Kjeld et al. 2018). However, contrary to Bae et al. (2003), we failed to report any between group FA differences. We propose that these discrepancies may be attributed to the fundamental differences in the two populations such as the breath-hold diving activities, the length of water exposures and/or the differences in water temperatures in which their breath-hold activities are being performed. A higher C:F_i_ and a lower R_95_ was observed in the type I muscle fibres of EBHD which supply a similar type I muscle FA when compared with the ND. This is an important finding as it entails an enhanced oxygen exchange rate capacity at the skeletal muscle tissue level due to a greater number of capillaries surrounding a similar muscle FA. However, it is currently difficult to quantify what effect these morphological characteristics may have on EBHD apnoeic capacities, as to date little is known about the balance between convective oxygen transport and the oxygen requirements of active skeletal muscle during voluntary breath-hold dives. Accordingly, future research should seek to examine in both EBHD and ND the blood flow regulation and skeletal muscle oxygen consumption during voluntary breath-hold dives.

The second novel aspect of this study was higher (27%) myoglobin content was observed in the EBHD type I muscle fibres when compared with the ND type I fibres. The higher myoglobin concentrations reported in the EBHD group might be the result of a training induced stimulus. Hypoxia coupled with skeletal muscle activation in both humans (Terrados et al. 1990), diving mammals and rodents has been documented to enhance myoglobin concentration in a muscle-specific manner (Dolar et al. 1999; Kanatous et al. 2008; Kanatous and Mammen 2010; Ponganis et al. 2010). Conversely, (1) hypoxic exposure and (2) skeletal muscle activation in normoxic conditions has been shown to impair these adaptive myoglobin responses (Jacobs et al. 1987; Terrados et al. 1990; Masuda et al. 1999). Thus, the higher myoglobin content observed in the type I muscle fibres of the EBHD group might be the result of a complex interplay between skeletal muscle activation and hypoxia.

Myoglobin is an oxygen-binding protein, thus when oxygen diffuses from the red blood cell across the sarcolemma, it binds to myoglobin. The myoglobin diffuses the oxygen to the mitochondria to meet the mitochondrial demand for oxygen, to produce energy in the form of adenosine triphosphate (Postnikova and Shekhovtsova 2013). Thus, myoglobin serves a central role in balancing intracellular hypoxia and aerobic metabolism in response to exercise (Postnikova and Shekhovtsova 2013). When skeletal muscle activity increases and the intramyocellular oxygen levels begin to decline as a result of an increased contractile activity (e.g. during dynamic apnoeas) (Overgaard et al. 2006), myoglobin helps to stabilise the oxygen levels inside muscle fibres by releasing its bound oxygen, making it available for aerobic metabolism (Hoppeler and Vogt 2001; Kanatous et al. 2009). As a result, the active skeletal muscle can rely mainly on stored oxygen to sustain aerobic metabolism, allowing for extended dive durations (Kooyman and Ponganis 1998; Polasek and Davis 2001). Thus, the greater myoglobin stores reported in the EBHD group might be an important adaptation to apnoeic diving, as a greater skeletal muscle oxygen reserve will be readily available to support aerobic metabolism during apnoeic durations. Therefore, our findings might provide a partial understanding to the lower post-apnoea blood acidosis and oxidative stress reported by Joulia et al (2002, 2003) in EBHD compared with ND.

Mitochondrial content was only higher in the type I muscle fibres (35%) when compared with type II fibres in the EBHD, whereas we did not observe any fibre-type group differences. The lack of mitochondrial content fibre-type difference in the ND is supported by Hoppeller (1986) findings who likewise reported a similar mitochondrial content across the muscle fibres of trained individuals (Saltin et al. 1977). Additionally, our findings are in agreement with Kjeld et al. (2018) who showed a similar citrate synthase maximal enzyme activity in EBHD with ND controls. Qualitative assessment of the relative myoglobin and mitochondrial protein distribution in muscle fibres revealed, a stronger fluorescence intensity and a homogenous distribution of myoglobin and mitochondria at the sub-sarcolemmal regions of type I muscle fibres in the EBHD compared with ND. We hypothesised that the homogenous distribution potentially reduces the intracellular diffusion distance between oxy-myoglobin and mitochondria. This novel observation in combination with the higher myoglobin concentrations reported in the EBHD type I fibres, may suggest a greater oxygen reserve being readily available to support a similar (when compared with the ND type I mitochondrial content) mitochondria respiration.

In conclusion, the present study highlighted (1) a similar splenic volume and mitochondrial content, (2) a higher capillary density, (3) a lower R_95_, (4) and a higher oxygen storage capacity in both blood (higher resting red blood cell count) and skeletal muscle tissue (higher myoglobin concentration) in EBHD compared with ND. Accordingly, future research should aim to assess the chronic responses of apnoeic training.

## Abbreviations

CC: Capillary contacts
CS: Capillary supply
CFPE index: Capillary-to-fibre perimeter exchange index
C:F: Capillary-to-fibre ratio
COXIV: Cytochrome oxidase IV
R_95_: Diffusion distance
EBHD: Elite breath-hold divers
FA: Fibre area
FITC: Fluorescein isothiocyanate-conjugated
ND: Non-divers
PBS: Phosphate buffered saline
SF: Sharing factor
UEA-I: Ulex Europaeus Agglutinin-I
